# Assessing the relationship between training load and injury in ultramarathon runners: a novel approach using Generalised Additive Models

**DOI:** 10.17159/2078-516X/2025/v37i1a20747

**Published:** 2025-07-15

**Authors:** TL Burgess, P Durand, K Buchholtz

**Affiliations:** 1 Division of Physiotherapy, Faculty of Health Sciences, University of Cape Town, Cape Town, South Africa; 2 Centre for Medical Ethics and Law, Faculty of Medicine and Health Sciences, Stellenbosch University, Cape Town, South Africa; 3 Department of Health, LUNEX University of Applied Sciences, Luxembourg, South Africa; 4 Luxembourg Health & Sport Sciences Research Institute A.s.b.l., 50, Avenue u Parc des Sports, 4671 Oberkorn Differdange, Luxembourg, South Africa

**Keywords:** running-related injuries, Comrades, acute-chronic workload

## Abstract

**Background:**

Ultramarathon running presents significant injury risks, and monitoring training loads may identify risk factors for injury. Injury surveillance studies are required to better assess injury prevalence and its relationship to training loads.

**Objectives:**

To determine the incidence and nature of running-related injuries and associated training loads in runners 12 weeks before and two weeks after the 2018 Comrades ultramarathon.

**Methods:**

One hundred and six participants were recruited. Their weekly injury and training load data (distance, duration, frequency and acute-chronic workload ratio) were obtained retrospectively over 14 weeks. The relationship between training load variables and injury risk was modelled using Generalised Additive Models.

**Results:**

The running-related injury incidence was 8/1000 hours. The overall injury proportion was 40%. The commonly injured structures were muscles (47%) followed by tendons (24%). Commonly reported anatomical areas of injury were the knee (26%) and hip (19%). Lower training load distance in the 12 weeks leading up to the race was linked to a higher risk of injury (p=0.02), primarily occurring during or after the race. Weekly training frequency and injury risk showed a significant heterogeneous relationship (p=0.02). The effect of the acute to chronic workload ratio on injury risk was minimal (p=0.3).

**Conclusion:**

Lower training loads were associated with a higher risk for injury, and the frequency of running training per week influenced injury risk. Insufficient training may not prepare the runners for the demands of the ultradistance race. Sudden changes in training load (evident in the acute training load measurements) appeared to have a minimal effect on injury risk. The non-linear relationship between several training load variables and injury risk can successfully be modelled using Generalised Additive Models, which may improve the accuracy of injury prediction modelling in ultramarathon runners.

Participation in ultramarathon running has seen exponential growth over recent years.[1,2] This may be attributed to the numerous health benefits, ease of access and affordability of the sport.[3] Conversely, ultramarathons have also been associated with a high prevalence of running-related injuries (RRIs), resulting in time loss from training and competition and socio-economic costs related to missing work due to injury or medical care-related costs.[1,4]

Previous studies have reported the prevalence of RRIs to range between 30% and 80%.[3,5] Across 13 studies, an RRI incidence rate of 2.5 to 33 injuries per 1000 hours of running exposure has been reported.[4] The wide range of prevalence and incidence are hypothesised to be related to a lack of standardisation between studies in injury recording and reporting methods. To address the lack of standardisation of epidemiological data, the International Olympic Committee (IOC) released a consensus statement with recommendations for researchers when reporting sport-related injuries.[6] Ongoing injury surveillance studies on ultramarathon runners that report epidemiological data according to recent recommendations have received global attention.

Risk factors for injury can be broadly categorised as either modifiable or non-modifiable.[7] Training load is a well-known modifiable risk factor for injury, showing positive or negative effects on RRIs.[8] Although research aimed at predicting the relationship between training load and injury risk in ultramarathon running specifically is limited, a systematic review on endurance sports reported a significant association between training distance, duration and frequency, and the incidence of injuries.[9] Therefore, appropriately modifying and managing various aspects of the training load may reduce the high injury rate reported.[7] The acute-to-chronic workload ratio (ACWR) is used globally as a prognostic tool in determining athletic performance, well-being and injury risk.[10,11] The ACWR compares athletes’ acute training load (one week) with the chronic training load (three to six weeks rolling average) to evaluate athletic preparedness.[9,11] The ACWR is calculated as (acute workload [km in the past 7 days])/(chronic workload [average of km per week over three to six weeks]). The researcher determines the number of weeks used for the rolling chronic workload based on the needs of the study.[11]

However, the calculation of the ACWR has been debated, with researchers arguing that the mathematical coupling of the chronic and acute load values is questionable.[10,12] Wang et al.[13] and Wang et al.[10] demonstrated that the relationship between the ACWR and injury risk is best represented when using an uncoupled ACWR in Generalised Additive Models.

Recent calls for better handling of non-linear data in sports medicine research prompted this [10,13,14,15]. Generalised Additive Models are automatic flexible statistical methods that may be used to identify and describe non-linear regression effects.[16] It has been proposed that modelling training load and injury risk using Generalised Additive Models might be better suited to model the flexible relationship across the diverse exposure range of training load and injury risk.[10,13]

The objectives of this study were to determine the incidence and nature (anatomical region, type, and time loss) of running-related injuries and their association with training loads in runners 12 weeks before and two weeks after the 2018 Comrades ultramarathon.

## Methods

### Study design

This study had a retrospective, cross-sectional design with repeated measures. One hundred and six participants were recruited, from whom weekly injury and training load data from 12 weeks before and two weeks after the 2018 Comrades ultramarathon were obtained retrospectively. Training load data were gathered from data stored on Global Positioning System (GPS) watches, and the participants reported injuries retrospectively.

### Setting and participants

Participants were recruited from eight running clubs and three community-organised road races situated in Pretoria, Johannesburg, and Midrand regions in South Africa. The Comrades ultramarathon is the largest in the world, providing a substantial platform for conducting a surveillance study on ultramarathon participants. In 2018, Comrades was a ‘down run’, and the approximately 90-kilometre (km) route covered a hilly course for the first 60 km, with the last 30 km featuring a predominantly downhill profile, which resulted in the ‘down-run’ title, in comparison to alternate years in the opposite direction ‘up-run’. The total elevation gain during the course is approximately 1095 meters (m), with the highest point at 823 m above sea level.

Both male and female ultramarathon runners older than 20 years (minimum age requirement for the race) were included in this study. Participants were only included if they had completed the 2018 Comrades ultramarathon and used GPS watches to track and store their training data.

### Data capturing and analysis

Two methods of data capturing were used:

*‘Face-to-face facilitation group,’* where the researcher conducted in-person data collection with each participant, or ‘*Online facilitation,’* via Zoom or WhatsApp calls. The participants completed the informed consent form, a baseline questionnaire on training history, and the injury logbook.

Descriptive characteristics of the participants’ demographics were described (mean, SD, 95% CI). Participants were allocated to the ‘injured’ group or ‘uninjured’ group and analysed using incidence percentages for the population, injured and non-injured groups (IBM Corp, USA. IBM SPSS Statistics for Macintosh, Version 25.0, 2017).

Training variables (distance, duration, frequency, and ACWR) were presented as cumulative 14-week and weekly averages. The ACWR was calculated by dividing the workload by km per week (acute) and the average of the preceding four weeks (chronic). An ACWR ratio of 1 may be interpreted as the runner running equivalent km in the current week as the average of the previous weeks. In contrast, a ratio below 1 means they have completed less training load during the current week than the previous average load. A ratio above 1 means that the runner completed more acute training load (in that current week) than the average of the previous weeks.[11]

An independent samples t-test was conducted to identify any significant differences in the training variables. A Mann–Whitney U test was used to compare the number of races. Cohen’s effect sizes were used to quantify the size of the difference. One way Analysis of Variance (ANOVA) and repeated measures ANOVA were performed to determine relationships between training variables and injury incidence over time.

To predict relationships between training load variables and injury risk over time, Generalised Additive Models were fitted in R (version 3.6.1, R Core Team, Austria (2019), https://www.R-project.org/) using the *mgcv1* package (version 1.8–31 for Generalized Additive Models).[13] Specifically, the relationship between training load variables (training distance, frequency, and the ACWR) and injury (dichotomised to yes/no) were modelled using Generalised Additive Models. Separate models were created for each training load variable (distance, frequency, and ACWR). All models specified a binomial distribution with a p-value <0.05 used to evaluate the significance of the effect predicted by the models. Similarly, Generalised Additive Models were fit to predict the relative risk for injury with training load variables; these required models to be calibrated as a Poisson distribution.

Relative risk (RR) with 95% CI was modelled in the Generalised Additive Models for training distance, frequency, and the ACWR. A RR of greater than 1 implied an increased risk for injury, while a RR of less than 1 indicated a decreased injury risk.

### Ethical considerations

The Faculty of Health Sciences Human Research Ethics Committee at the University of Cape Town (HREC/REF: 356/2018) gave ethical approval to conduct the study, and participants gave informed consent before their inclusion.

## Results

### Participants’ characteristics

One hundred and fifty-six participants were recruited for this study. Thereafter, 50 participants were excluded based on the exclusion criteria (incomplete data), resulting in a sample of 106 participants. Descriptive characteristics are presented in Table 1.

A significant difference was found between the injured (n=43) and uninjured (n=63) groups in the number of Comrades

Ultramarathons completed (U_(n=42,n=64)_=991.5; z=−2.2; p=0.03) with the uninjured group having completed more Comrades ultramarathons as shown in Table 1.

### Injury profiles

The prevalence of previous RRIs before the study period was 74% (n=78). In the injured group (n=43), 84% had a history of RRIs, compared to 67% in the uninjured group (n=63). A significant difference between the injured and uninjured groups (*X*^2^=5.265, p=0.02) was found. Participants who reported a previous RRI were more likely to sustain an injury during the study compared to participants who had no history of previous injury.

Table 2 presents the total number of injuries (n=58) sustained during this study. Based on the IOC definitions, the injuries are classified as index, subsequent, exacerbation, or recurrent.[6] Exacerbations were not included in the total count as the definition implies that the injury occurred before the start of the study and had not fully healed yet.

### Weekly injury prevalence

The highest weekly injury prevalence (13.2%) occurred during week 13, which was the week following the Comrades ultramarathon. The second highest injury prevalence (10.4%) was recorded in the first week of the study which was 12 weeks before the race. The average weekly prevalence of RRIs was 5% (mean 4.9±3.4%).

### Anatomical region and types of injuries

Muscles (47%) followed by injuries to tendons (24%) with an incidence of 4 and 2.5 injuries/1000 hours of running participation respectively were the most common structures injured. The knee (26%) followed by the hip (19%), and the foot (14%) were the most common anatomical structures reported as shown in Table 3.

### Training load profiles in relation to running-related injuries

A significant difference in average weekly training frequency (sessions.wk^−1^) (t=3.27, df=1467, p=0.001) and distance (km.wk^−1^) (t=2.71, df=1468, p=0.007) was found between the two groups as shown in Table 4. The injured group had a lower average frequency and distance (km.wk^−1^) of running per week.

### Training distance, duration and frequency

**Fig. 1:**
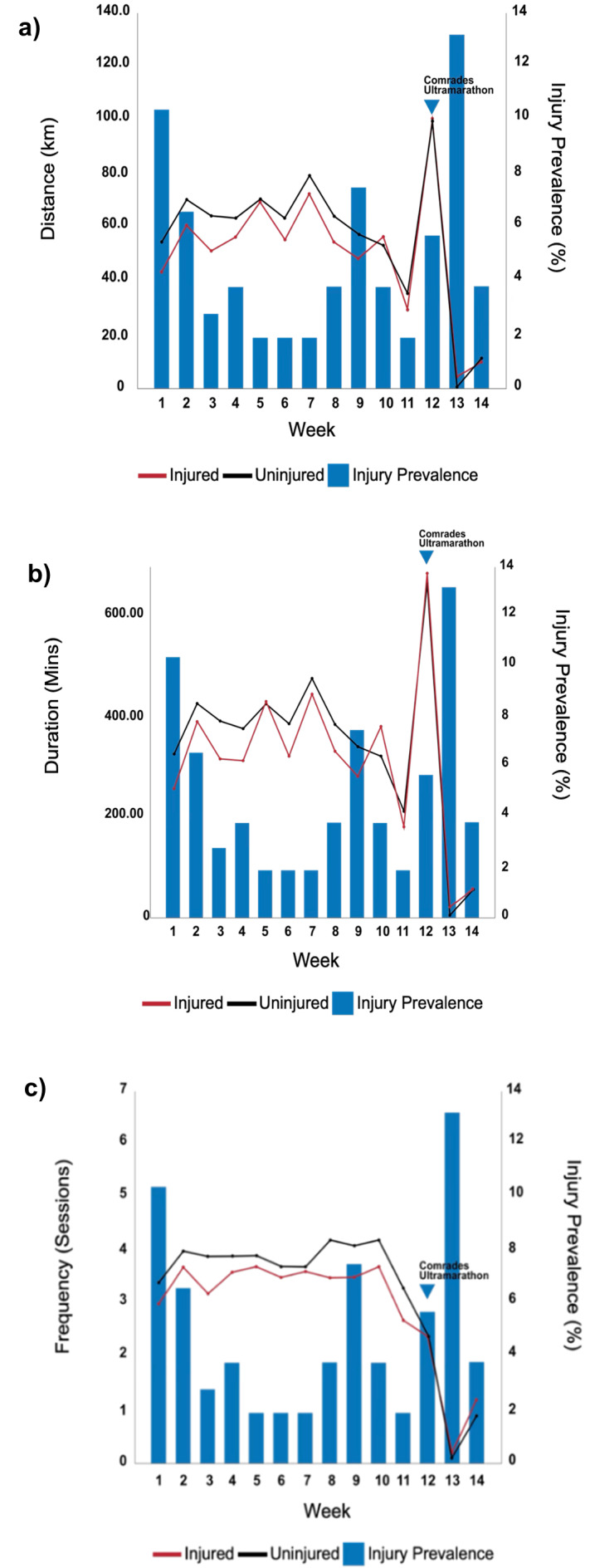
Average weekly training (a) distance, (b) duration, (c) frequency between the injured and uninjured group over the 14-week study period in kilometres. The average weekly prevalence of injuries (%) is presented.

The weekly training distance of the injured group was consistently lower than the uninjured group (F_(9, 926)_=12.35; p=0.000004) in the first 11 weeks before the Comrades ultramarathon (Figure 1).

As shown in Figure 1, the injured group had a lower average weekly training duration than the uninjured group (F_(7, 101)_=7; p=0.004) in the first 11 weeks before the Comrades ultramarathon. The injured group consistently trained less often per week than the uninjured group (F_(13, 1326)_=88.1; p=0.00001) over the 14-week period presented in Figure 1.

### Acute: chronic workload ratio

Descriptive statistics for weekly average ACWR showed no significant difference between the injured and uninjured groups (Supplementary data).

### Predicting the relationship between training load parameters and RRI risk

#### a) The relationship between the ACWR and RRI risk

**Fig. 2:**
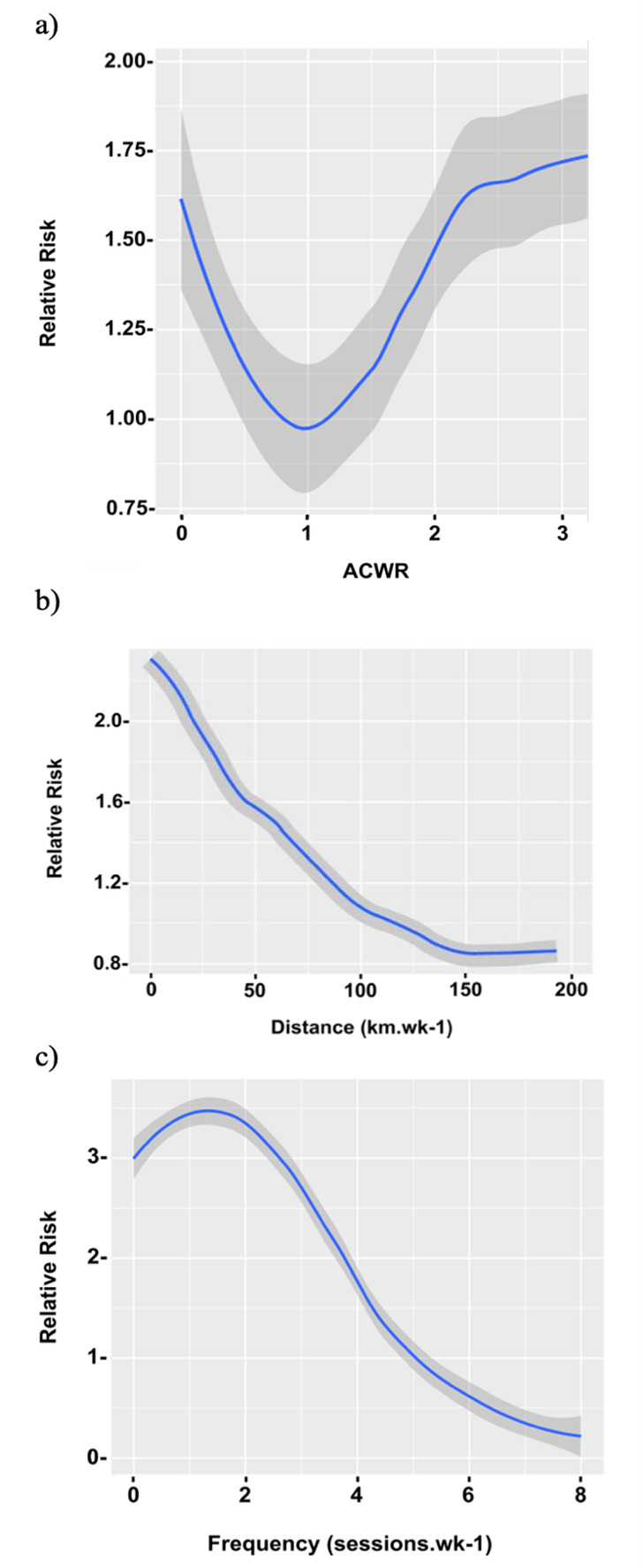
Generalised Additive Models illustrating the relationship between a) the 10-week acute-to-chronic workload ratio (ACWR) and injury risk in ultramarathon runners. b) the 14-week training load variable of distance (km.wk^−1^) and injury in ultramarathon runners, and c) the 14-week training load variable of frequency of training (sessions.wk^−1^) and injury in ultramarathon runners. The blue line represents the model with 95% CI in the shaded area. The relative risk of injury is shown on the y-axis.

A Generalised Additive Model was used to model the interaction between the ACWR and injury risk. The ACWR had no significant effect on injury risk (R^2^=0.074, df=1.00, p=0.3), although the Generalised Additive Model was able to model the non-linear u-shaped relationship with minimal increases in RR as shown in Figure 2.

Data with ACWR >2.5 was limited in our study, resulting in greater uncertainty of the model over the ACWR range of 2.5. A relationship between lower and higher ACWRs and increased risk is shown by the Generalised Additive Model in Figure 2a. However, the magnitude of increased injury risk in this model was minimal with a maximum increase in RR of 0.7 which was not statistically significant.

#### b) The relationship between weekly training distance and RRI risk

The Generalised Additive Model depicting the relationship between the training load variable of training distance (km.wk^1^) and injury risk indicated a more linear relationship. A significant inverse linear relationship was found (R^2^=−2.311, df=1165, p=0.02).

A stable injury risk was shown when the weekly training distance was just over 100 km.wk^−1^ (RR _Distance=107_=1, p<0.05). The RR was the highest at the lowest training distance (RR _Distance=0_=2.3, p<0.05).

A decrease in relative risk was then observed as the training distance increased, reaching the lowest relative risk at a training distance of just over 150 km.wk^−1^ (RR _Distance=160_=0.8, p<0.05) as presented in Figure 2b.

#### c) The relationship between weekly training frequency and RRI risk

The Generalised Additive Model predicting the relationship between injury and frequency of running training per week showed a significant effect (R^2^=3.293, df=2.62, p=0.02).

The RR of injury was shown to be the highest with a training frequency of <2 sessions per week with a relative risk of injury just under 3 (RR _Frequency=0_=3, p<0.05; RR _frequency=2_=3.3, p<0.05). With a training frequency of >2 sessions.wk^−1^ the RR for injury declines, until a stable injury risk was shown with a weekly training frequency of five sessions per week (RR _Frequency=5_=1 p<0.05). Training more than five sessions per week was shown to be protective against injury. The lowest RR is shown at a training frequency of eight sessions.wk^−1^ (RR _Frequency=8_=0.25, p<0.05) as shown in Figure 2c

## Discussion

In this study, we aimed to investigate the incidence and nature of RRIs and their association with training loads in runners 12 weeks before and two weeks after the 2018 Comrades ultramarathon. A wide range in prevalence and incidence of RRIs across studies exists which is likely due to differences in injury definitions, sample size, and the specific population studied.[2–5,7,17,18] We observed a higher injury rate with an overall incidence of 8 injuries per 1000 hours compared to 5 injuries per 1000 hours of running training reported by Craddock et al.[12], and lower than the average injury rates reported by Hoffman and Fogard[17] and Hoffman and Krishnan [5]. The higher injury rate reported in the present study may be explained by the longer event distance the participants trained for compared to other studies.[12,19]

Two retrospective studies on ultramarathon runners by Hoffman and Fogard[17] and Hoffman and Krishnan[5] reported a higher RRI proportion prevalence of 65% and 52%, respectively, compared to 40% in this study. These studies had a larger sample size, which may have resulted in greater statistical power. The authors also assessed runners over 12 months, while our study evaluated only for injuries sustained during 14 weeks.

The most prevalent type of structures injured were muscles (3.7/1000h) followed by injuries to tendons (2.5/1000h). Muscle injuries had a higher incidence in the quadriceps, calf, and hip region, while tendon injuries had a higher incidence in the hip and knee region, similar to related studies.[5,12,17]

The knee (26%) was reported as the most injured anatomical area, similar to Craddock et al.[12] with 19%, and in line with numerous studies ranging between 7% and 50%.[4,18,19] Due to the greater burden that injuries to the knee and foot have on ultramarathon runners, as reported by this study, further research should focus on possible aetiological factors regarding the development of knee and foot injuries in ultramarathon runners.

The severity of injuries differed according to the type of structure injured, with tendon injuries resulting in the most time loss. Compared to related studies, we found the median injury severity according to time-loss in days to be four days, aligned with Hoffman and Krishnan[5], who reported a median of five days’ time-loss.

The relationship between training load and injury risk has long been proposed to be non-linear.[20] Recently, there has been a call for better handling non-linearity in sports medicine research.[14,15] Statistical methods commonly used in training load research have been criticised for not accounting for repeated measures.[13–15] This has been addressed by using General Linear Models in certain cases and discretising the training load parameters for analysis.[13]

Generalised Linear Models are not ideally suited as they assume that the relationship between the training load variables (external training load and ACWR) and the outcome variable (injury status) is constant over the exposure range.[13] It has also been argued that data discretisation is inappropriate for continuous data and can result in inflated discovery rates or negative results.[15] It has been recommended that the methods used in future research should be able to explore and model non-linearity to discover the relationship and should not constrain it.[14] Further, it has been proposed that modelling training load and injury risk in GAMs might be better suited to model the flexible relationship across the diverse exposure range of training load and injury risk.[10,13]

The present study modelled training load variables using traditional statistical modelling methods and Generalised Additive Models. The modelled relationship between injury risk and weekly training load confirms our finding that a lower weekly training mileage is associated with an increased injury risk, which is supported in related literature.[12,18] A significant inverse linear relationship between a lower training distance and greater injury risk was reported using a Generalised Additive Model. The relative risk for injury gradually increased as the weekly mileage decreased, reaching a two-fold increase in injury risk in participants who trained less than 25km per week compared to participants who trained 100km per week. Craddock et al.[12] reported that a weekly training distance of less than 30km per week was a risk factor for injury in ultramarathon and marathon participants, respectively. It is reasonable to argue that participants might have a more cautious approach to training following an injury. This could have led to a reduction in the weekly training load as a result of the injury.

A stable injury risk (neither an increase nor decrease in risk) was reported when the weekly training distance was slightly over 100km per week. A maximal reduction in injury risk was found when the training distance was approximately 150km per week. Research has suggested that an upper limit of training volume exists, at which point an increase in injury risk transpires.[3,8] We found that such an upper limit may be much higher in the population of ultramarathon runners, as a reduced injury risk with a weekly training distance as high as 180km per week was predicted using the Generalised Additive Model.

Research on training load injury has been criticised for numerous conceptual and methodological pitfalls.[21] More specifically, the overreliance on the training load metric of ACWR in related literature has been criticised.[21] The lack of a conceptual framework to support the theoretical mechanisms that might explain the influence of ACWR on health and performance outcomes has been noted.

Using the Generalised Additive Model, our study modelled the relationship between injury risk and ACWR and illustrated a heterogeneous relationship. The effect of weekly ACWR on injury risk was not significant in our study population. To our knowledge, the present study is the first to use Generalised Additive Models to predict the relationship between injury risk and the ACWR in ultramarathon runners. We cannot compare the present findings with the current literature. Only two other studies used Generalised Additive Models to model ACWR data with injury risk.[10,13] In these studies, injury risk was investigated in children based on changes in physical activity over time.[10,13] The ACWR with injury risk modelled as a Generalised Additive Model was a better predictor of injury risk when compared to the General Linear Model.[10,13]

The Generalised Additive Models used in the present study illustrate a U-shaped relationship between injury risk and ACWR. The Generalised Additive Model predicted a stable injury risk with an ACWR of between ~0.8 and 1.1. Both an ACWR of <0.8 and >1.1 resulted in an increased RR for injury, with the highest RR at an ACWR=0 and ACWR=2.2, similar to findings by Wang et al.[13]. Our model predicted that the increase in injury risk was minimal with the increase in ACWR, much lower than what is predicted by the IOC’s model.[20]

Our findings using a Generalised Additive Model closely resembled the proposed model by the IOC on the relationship between changes in training load and injury risk in athletes.[20] Due to the non-linear nature of the proposed relationship between ACWR and injury risk, a Generalised Additive Model may provide better insight into the predictive nature of ACWR on injury risk in athletes in future studies.

Regarding the practical implications of these findings, there seems to be a clear indication that insufficient training/low training load during the final 12 weeks before an ultramarathon increases the risk of sustaining an injury. While other possible confounding factors, like speed, were not considered (many speed sessions may be performed at higher velocities than longer duration runs), the findings do support previous hypotheses that low training loads may not sufficiently prepare runners for the demands of the long duration and distance in ultramarathons.

### Limitations

While a modest sample size (n=106) was used in this study, similar studies included a participant range between 32 and 873.[9] Self-reporting of the type of injury may be unreliable as it depends on participants’ subjective understanding of their injury. At the same time, the reporting of anatomical areas seems to be more reliable.[5]

The study’s retrospective design may have led to recall bias of when an injury occurred. Individuals were recruited within five months after the 2018 Comrades ultramarathon to minimise recall bias. Data obtained retrospectively within 12 months has a minimal recall bias of approximately 12%.[22] In addition, the researchers facilitated the completion of the questionnaires either in person or online to clarify any questions that arose which would have prevented the participants from completing the questionnaires due to confusion. Using the objective data from the GPS devices was an attempt to reduce the risk of recall bias related to the distance, duration and frequency. Still, it resulted in other potential biases due to the economic burden of such devices in a country like South Africa with significant socioeconomic disparities.

Our study is unique because it obtained training data stored on GPS watches. All runners were required to have a GPS watch to participate in this study. However, we recognise that GPS watches can be costly and may not be accessible to many runners in South Africa, potentially skewing the eligibility of numerous runners who would otherwise have been suitable for the study and contributing to a more diverse population. Future studies should include GPS watches as a standard line item for research funding to ensure research is accessible by a representative sample of runners.

## Conclusion

The overall injury incidence reported in this study was comparable to that of the existing literature. The proportional prevalence of running-related injuries was lower than previously reported. Lower training loads were associated with a higher risk for RRIs in ultramarathon runners. The frequency of running training per week substantially influenced injury risk. Sudden changes in training load, as calculated by the ACWR, appeared to have a minimal effect on injury risk in the ultramarathon runners’ population.

The non-linear relationship between several training load variables and injury risk can successfully be modelled with Generalised Additive Models. Future research is required to establish whether the predicted parameters of training load variables may assist in preventing injury in ultramarathon runners in well-designed randomised controlled trials.

## Supplementary Information



## Figures and Tables

**Table 1:** Descriptive characteristics and endurance running participation history of participants grouped in total, injured and uninjured groups

	Total (n=106)	Injured (n=43)	Uninjured (n=63)	t-value	p-value
Age (years)	40.5±7.9	40±6.5	40.8±8.8	−0.54	0.59
Height (cm)	172.2±11.1	170.4±11.3	173.4±10.9	−1.36	0.18
Mass (kg)	71.3±13.0	71.5±11.4	71.1±14.0	0.16	0.87
Participation in endurance running (years)	9.7±7.5	9.7±6.8	9.7±7.9	0.001	1.00
Ultramarathons completed (n)	8±12	7±10	9±13	1085 (U-value)	0.09
Comrades ultramarathons completed (n)	3±4	4±2	5±4	992 (U-value)	0.03*

* p<0.05.

Data are expressed as mean ± standard deviation (SD).

**Table 2:** Summary of injury count (n), prevalence (%), incidence (/1000h) and severity in time loss

	Injuries n	Prevalence (%)	Incidence (/1000 hours)	Time-loss (days) Median (range)
Index injury	43	40.7%	6.3	4 (0–25)
Subsequent injury	12	9.4%	1.8	3 (0–9)
Exacerbation injury	9	5.7%	1.3	3 (0–9)
Recurrent injury	3	2.8%	0.4	2 (0–4)

**Table 3:** Injury distribution, incidence (n/1000 hours) and time-loss (days) of all recorded injuries

Injury description – Anatomical region / type of structure	Injuries n	Incidence Injuries/1000 hours	Time-loss Days (95% CI)

**Total injuries by structure type**			
Muscle	27	3.7	2.9 (0–9)
Tendon	14	2.5	4.5 (0–25)
Ligament	3	0.7	3.5 (2–5)
Joint	2	0.3	3 (0–6)
Bone	2	0.4	1 (0–2)
Nerve	3	0.4	4 (3–5)
Other	8	1.5	3.3 (0–9)

**Injuries by region and structure type**			

**Back**	**2**	**0.3**	**4 (2–6)**
Muscle	1	0.1	2 (2–2)
Joint	1	0.1	6 (6–6)

**Hip**	**11**	**1.6**	**2.6 (0–6)**
Muscle	4	0.6	1.3 (0–4)
Tendon	4	0.6	4.3 (0–6)
Bone	1	0.1	2 (2–2)
Nerve	1	0.1	5 (5–5)
Do not know	1	0.1	2 (2–2)

**Quadriceps (front of thigh)**	**6**	**0.9**	**4.9 (2–13)**
Muscle	6	0.9	4.8 (2–11)
Tendon	1	0.1	2 (2–2)

**Hamstrings (back of thigh)**	**3**	**0.4**	**3 (2–4)**
Muscle	3	0.4	3 (2–4)

**Groin**	**2**	**0.3**	**7.5 (6–9)**
Tendon	2	0.3	7.5 (6–9)

**Knee**	**15**	**2.2**	**3 (0–9)**
Muscle	3	0.4	3 (0–5)
Joint	1	0.1	0 (0–0)
Tendon	3	0.4	1.5 (0–3)
Ligament	2	0.3	3 (2–4)
Nerve	2	0.3	3.5 (3–4)
ITB	3	0.4	15 (9–20)
Do not Know	1	0.1	0 (0–0)

**Calf**	**7**	**1**	**2.8 (0–4)**
Muscle	6	0.7	2.5 (0–4)
Tendon	1	0.1	4 (4–4)

**Shin**	**1**	**0.1**	**0 (0–0)**
Muscle	1	0.1	0 (0–0)

**Ankle**	**3**	**0.4**	**5 (4–6)**
Muscle	1	0.1	4 (4–4)
Tendon	1	0.1	6 (6–6)
Ligament	1	0.1	5 (5–5)

**Foot**	**8**	**1.2**	**5.7 (0–25)**
Muscle	2	0.3	4.5 (4–5)
Tendon	2	0.3	9 (3–15)
Bone	1	0.1	0 (0–0)
Skin	1	0.1	5 (5–5)
Plantar Fasciitis	1	0.1	2 (2–2)
Do not know	1	0.1	3 (3–3)

**Table 4:** Training load variables (average duration, average distance, average frequency, average (ACWR) during the 14-week study period

	Total (n=106)	Injured (n=42)	Uninjured (n=64)	t-value	df	p-value	Cohen’s d effect size
Average Frequency (sessions.wk^−1^)	3.0±2.0	2.9±1.9	3.3±1.9	3.27	1467	0.001*	0.17
Average Distance (km.wk^−1)^	54±37	51±39	56±36	2.71	1468	0.007*	0.14
Average Duration (min.wk^−1^)	335±239	321±264	345±219	1.91	1467	0.06	0.10
Average ACWR (AU)	0.9±0.6	0.9±0.6	0.9±0.6	−0.72	1163	0.53	−0.04

* p<0.05.

Data presented in total, injured and uninjured groups and expressed as mean±standard deviation (SD); ACWR, acute-to-chronic workload ratio
