# Supplementary material for: Assessing the relationship between training load and injury in ultramarathon runners: a novel approach using Generalised Additive Models
Source: S Afr J Sports Med. 2025 Jul 15;37(1):v37i1a20747. doi: 10.17159/2078-516X/2025/v37i1a20747 (PMC12327882; doi:10.17159/2078-516X/2025/v37i1a20747)
Supplement: Supplementary file 1 [file 2078-516X-37-v37i1a20747-s001.pdf]

Assessing the relationship between training load and injury in ultramarathon runners: a novel approach using Generalised Additive Models

Supplementary Table: Acute:chronic workload ratios (AU) over the 14-week study period. Data are presented in total, injured and uninjured groups and expressed as mean ± standard deviation (SD)

Week	Total	Injured	Uninjured	t-value	p-value
**5**	1.1	1.2±0.6	1.1±0.3	-1.843	0.07
**6**	0.9	0.9±0.4	1.0±0.4	1.132	0.3
**7**	1.2	1.2±0.4	1.2±0.4	0.233	0.8
**8**	0.9	0.9±0.5	0.9±0.4	-0.009	1
**9**	0.8	0.8±0.4	0.8±0.3	0.695	0.5
**10**	0.9	0.9±0.4	0.8±0.3	-0.816	0.4
**11**	0.6	0.6±0.4	0.7±0.3	0.882	0.4
**12**	1.8	1.9±0.6	1.8±0.6	-1.331	0.2
**13**	0.0	0±0.1	0.0±0.1	-1.025	0.3
**14**	0.2	0.2±0.3	0.2±0.3	-0.365	0.7
